# Clinical and molecular spectrum of 46,XY disorders of sex development that harbour *MAMLD1* variations: case series and review of literature

**DOI:** 10.1186/s13023-020-01459-9

**Published:** 2020-07-20

**Authors:** Lele Li, Chang Su, Lijun Fan, Fenqi Gao, Xuejun Liang, Chunxiu Gong

**Affiliations:** Department of Endocrinology, Genetics, Metabolism and Adolescent Medicine, Beijing Children’s Hospital, the Capital Medical University, National Center for Children’s Health, 56# Nan Lishi Rd, West District, Beijing, 100045 P. R. China

**Keywords:** *MAMLD1*, Hypospadias, Disorders of sex development

## Abstract

**Background:**

*Mastermind-like domain-containing 1* (*MAMLD1*) has previously been identified as a causative gene for “46,XY Disorders of Sex Development (DSD)”. Recently, there has been some controversy regarding the causative role of *MAMLD1* variations in DSDs. Here we describe a clinical series and review the reported cases to evaluate the role of *MAMLD1* variants in children with 46,XY DSD. Cases of 46,XY DSD harbouring *MAMLD1* variants from unrelated families were recruited from the Beijing Children’s Hospital in China (*N* = 10) or identified through a literature search (*N* = 26). The clinical manifestations and genetic variants of all the patients were evaluated.

**Results:**

Hypospadias was the most prevalent phenotype among our 10 cases (8 out of 10 cases) and in all the previously reported ones. Central precocious puberty and isolated micropenis were observed for the first time. Among the 10 cases, nine variants were identified, including three nonsense (p.R356X, p.Q152X, and p.Q124X) and six missense (p.P334S, p.S662R, p.A421P,p.T992I, p.P542S, and p.R927L) variants. In silico analysis showed that the variants p.P334S, p.P542S, p.S662R, and p.R927Lmight lead to drastic changes in the interaction force of the amino acid chain and the flexibility of the spatial structure, and such changes may affect protein function.

**Conclusion:**

Patients with 46,XY DSD harbouring *MAMLD1*variants manifest a broad spectrum of phenotypes and mostly present with hypospadias. The six novel variants reported here enrich the mutation database and contribute to our understanding of the pathogenesis of 46,XY DSD.

## Introduction

Disorders of Sex Development (DSD) are congenital conditions associated with atypical chromosomal, gonadal, or anatomical sex [1]. DSD have been divided into three groups, sex chromosome DSD; 46,XX DSD; and 46,XY DSD [2]. 46,XY DSDs refer to a wide range of conditions with diverse features and pathophysiology. Affected patients present with the 46,XY karyotype, but their gonads and phenotypic genders are usually inconsistent with their genetic sexes. Its pathogenesis is complex, with both genetic and environmental contributions. Although various mutations in multiple genes, including *SRY*, *NR5A1(SF1)*, *WT1*, *SOX9*, *DAX1(NR0B1)*, *DHH*, *WNT4*, *DMRT1/2*, *ATRX*, *MAMLD1*, and *MAP 3K1*have been identified to be involved in the differentiation of the testis, the genetic cause(s)underlying~ 50% of 46,XY DSD cases are still elusive [3].

The *mastermind-like domain-containing 1*(*MAMLD1*) gene, previously known as *Chromosome X open reading frame 6* (*CXorf6*) or *F18* (OMIM# 300120), was first identified in two patients with myotubular myopathy and male hypogenitalism, who were found to harbour a deletion on chromosome Xq28 [4, 5]. Although *MAMLD1* is 70 kilobase long and contains 12 exons, only 7 of them have been validated to date [6]. Owing to in-frame alternative splicing, exons 3–6 encode two proteins of 701 and 660 amino acids, respectively, depending on whether the transcript includes or excludes exon 4. PCR-based human cDNA library screening has revealed ubiquitous expression of both splice variants, with the larger variant being the dominant form [7].

*MAMLD1*is expressed in foetal Sertoli and Leydig cells around the critical period for sex development, and the transient knockdown of *MAMLD1*significantly reduces testosterone (T) production in mouse Leydig tumour cells [8, 9]. *MAMLD1* is also co-expressed with steroidogenic factor-1 (*NR5A-1*), which is involved in testicular differentiation as a modulator of gene transcription. MAMLD1 transactivates the non-canonical-Notch–targeted *Hes3* promoter [8]. *Hes3* regulates cell differentiation and proliferation during embryonic development [9]. Taken together, *MAMLD1* has been identified as a causative gene for 46,XY DSDs [8, 10].

To date, approximately 20 *MAMLD1* sequence variations have been documented in the Human Gene Mutation Database and described in patients who have 46,XY DSDs, mostly presenting with hypospadias. However, there is some controversy regarding the causative role of *MAMLD1*variations in sex development. Several *MAMLD1* variations have exhibited wild-type (WT) activity in functional studies [11], and MAMLD1-knockout male mice present with normal genitalia and reproduction [12]. Recently, oligogenic disease has been proposed based on the observation that the broad phenotypes of DSDs in *MAMLD1* patients might involve multiple genetic variations contributing to the complex network of sexual development [13].

In this study, we described a clinical series of 46,XY DSDs and reviewed the reported cases to demonstrate the clinical features, hormonal profiles, and molecular characteristics of such patients. In addition, we carried out in silico analysis to evaluate the pathogenicity of *MAMLD1* variants in children with 46,XY DSD.

## Methods

### Subjects

Patients with 46,XY DSD admitted to the Beijing Children’s Hospital over the past 5 years were recruited for this study. These patients were subjected to next-generation sequencing (NGS) using a gene panel, or whole-exome sequencing (WES) that coveredthe *MAMLD1* variants.

### Case review

Using the keywords “46,XY DSD”, “MAMLD1”, “CXorf6”, and “F18”, we searched for published reports on confirmed 46,XY DSD cases with *MAMLD1* variations in the literature databases PubMed, Ovid Medline, Springer, CBMD, Wanfang, and CNKI, and the documented clinical data and genetic test results of the matched cases were compared with those of our cases.

### Clinical information

For the cases from our centre, the clinical information was obtained from medical records. Two experienced paediatric endocrinologists performed physical examinations and assessments. The information included, but not limited to, was the age at visit, social gender, chief complaint, family history, bone age, birth length, birth weight, gestational age, history of gestation, penile length, testis size, testis position, location of urethral meatus, scrotal appearance, electrolyte levels, and liver and kidney functions. Pituitary hormones were measured, and ultrasound or magnetic resonance imaging was used to examine the adrenal glands, pelvic gonads, and Müllerian duct structures of the patients.

The basal and stimulated luteinizing hormone (LH) and follicle-stimulating hormone (FSH) levels after gonadotropin-releasing hormone (GnRH, 100 μg/m^2^) administration in addition to the basal and stimulated plasma T levels after human chorionic gonadotropin (hCG), anti-Müllerian hormone (AMH), and inhibin-B (INHB) administrations were determined. Adrenal function was evaluated by measuring the serum cortisol, plasma adrenocorticotropic hormone (ACTH), 17-hydroxyprogesterone, and dehydroepiandrosterone sulfate levels. ACTH stimulation test was also performed in patient#4.For the hCG standard stimulation test, an injection of 1500 U hCG per day was administered for four consecutive days, and peripheral blood samples were collected 12 h after the last injection T, FSH, LH, ACTH, and cortisol levels were evaluated using the radioimmunoassay method (MAGLUMI®2000). AMH and INHB levels were evaluated using electrochemiluminescence and ELISA, respectively.

For the previously reported cases, the clinical presentations, hormone profiles, genetic variations, and other supplementary information were based on the comprehensive collection and coordination of academic literature.

### Molecular analysis

NGS using a DSD gene panel or WES was performed to detect *MAMLD1* variants. Genomic DNAs were extracted from the peripheral blood samples of the probands and their parents and sent to a qualified domestic company for commercial sequencing (Chigene Translational Medicine Research Center Co., Ltd.; Beijing Kangso Medical Laboratory Zhongguancun Huakang Gene Institute). The prepared libraries were sequenced at an average depth of more than 100× on the high-throughput NGS platforms, for example, Illumina HiSeq 2500 or HiSeq X Ten (Illumina, San Diego, CA, USA) according to the manufacturer’s instructions. The raw sequencing data underwent quality control via NGSQCToolkit33 and were filtered to remove poor quality reads, followed by alignment to the reference human genome sequence using BWA software. After excluding the duplicated reads and performing statistical analysis on the remaining reads, variants were called by using GATK software. The called variants were annotated based on public databases for mutation records and population frequency, such as HGMD, Clinvar, Exome Sequencing Project, 1000 Genomes Project, gnomAD, etc., and their deleterious effects were predicted in silico. Candidate variants were further confirmed by Sanger sequencing. The parents were also examined for the variants to determine whether the variants were inherited or de novo. Finally, the candidate variants were evaluated and classified according to the American College of Medical Genetics and Genomics (ACMG) and the Association for Molecular Pathology (AMP) standards guidelines. The reference sequence of the *MAMLD1* gene was NM_001177465.3.

### In silico analysis

MAMLD1 is associated with the family of mastermind-like proteins and associated diseases, with no available 3-D structural information. The available homologous template has a sequence similarity of 30–40% with the amino acid residues severely lost after modelling. Further, we failed to find a suitable template covering the unreadable amino acid residues by another homologous modelling mode (blastp + multi-template using PDB database). Finally, the sequences of WT and variant MAMLD1 were submitted to the I-TASSER server by the automatic threading method for building a model (https://zhanglab.ccmb.med.umich.edu/I-TASSER/) [14–16], with a C-score of − 0.30. Protein structural models for MAMLD1 were performed with PyMOL.

The research was approved by the Ethics Committee of the Beijing Children’s Hospital, Capital Medical University. Written informed consent was obtained from all the patients or their legal guardians. This study has been conducted in accordance with the principles of the Declaration of Helsinki.

## Results

Among the 590 recruited patients, 359 (60.85%) were found to have the disease-causing genetic variations associated with their clinical features. Of these, 10 cases (1.7%, patients #1–10) from unrelated families were identified to have mutations in the *MAMLD1* gene. From the literature search, 26 cases (patients #11–36) with *MAMLD1*-associated 46,XY DSD were identified.

### Clinical features

**Fig. 1:**
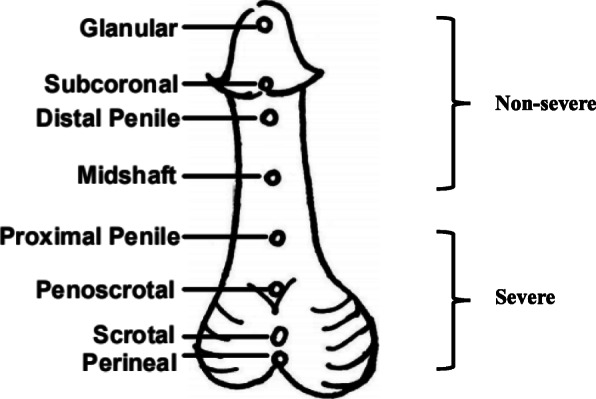
Clinical severity ranged from subcoronal to perineal. Patients were characterized as “severe” and “non-severe” groups

The clinical features of the 10 Chinese patients displaying *MAMLD1* gene mutations are summarized in Table 1, and their ages at first visit were all under 3 years; only patient #5 had a family history of hypospadias. Based on the position of the urethral meatus (Fig. 1), seven patients (patients #1–3, #6–8, and #10) had severe hypospadias, whereas one patient (patient#5) was classified as having a mild type of hypospadias. In addition to the genital malformation, patient #1 also showed macrocephaly, stunned forehead, and cupped ears. Patient #4 had normal male external genitalia at birth; however, he was admitted to our hospital because of premature secondary sexual characteristics, increased penis size, growing beard, and pubic hair at the age of 1 year. In addition, although the patient showed elevated LH, FSH, and T levels, which supported the precocious puberty, his T level later on decreased, and he displayed hypergonadotropic hypogonadism. According to the ultrasound results, none of the patients had the Müllerian structures, whereas the Wolffian and adrenal structures were normal.

**Table 1:** Clinical findings, endocrine parameters and genetic results of the 10 Chinese 46, XY patients with *MAMLD1* variants

Patient	Visit Age	Phenotype				Serum hormone values							***MAMLD1*** variants			
		Hypos- padias	Micro- penis	Crytor- chidism	Urethral Meatus	LH, IU/L	FSH, IU/L	T/T after HCG, ng/dl	INHB, pg/ml^**a**^	AMH, ng/ml	ACTH, pg/ml	Cortisol, μg/dl	Nucleotide Change	Amino AcidChange	Variant Type	ACMG
**1**	1.3 yr	+	+	**–**	Penoscrotal	<0.10	1.14	<20.0/257	158	>23	32.9	9.57	c.1986C>G	p.S662R	Missense	VUS
**2**	4 mo	**+**	**+**	**–**	Scrotal	0.16	0.18	<20.0/564.0	NA	NA	50.5	14.8	c.1066C>T	p.R356X	Nonsense	P
**3**	4 mo	**+**	**+**	**–**	Scrotal	6.63	3.16	30.9/744	123.8	>23	305/35.7	23.1	c.1066C>T	p.R356X	Nonsense	P
**4**	2.25 yr	Penis enlargement; Premature puberty			Normal	16.86^b^/0.3^c^/13.6^d^	6.78^b^/0.78^c^/10.7^d^	36.43^b^/<20.0^c^	237.64	>23	33.9^c^	7.58^c^/37.6^e^	c.1261G>C	p.A421P	Missense	VUS
**5**	3 mo	+	+	**–**	Subcoronal	6.45	3.66	163	89.5	>23	24.9	10.5	c.454C>T	p.Q152X	Nonsense	P
**6**	3 mo	**+**	**+**	**+**	Penoscrotal	0.189	4.7	<20.0/257.9	128.83	122	88.12/17.0^f^	10.2/13.50^f^	c.1624C>T	p.P542S	Missense	LB
**7**	3 yr	**+**	**+**	**+**	Perineal	<0.10	3.97	<20.0/89.45	NA	NA	43.6	10.7	c.1000C > T	p.P334S	Missense	LP
**8**	3 mo	**+**	**–**	**–**	Perineal	1.27	1.32	77.3/435	291.91	>23	120	6.18	c.370C > T	p.Q124X	Nonsense	P
**9**	2.5 yr	**–**	**+**	**–**	Normal	<0.10	0.59	135	125.3/309	>23	38.7	7.4	c.2975C>T	p.T992I	Missense	VUS
**10**	1 yr	+	**–**	**+**	Perineal	0.16	0.74	<20.0	NA	NA	NA	NA	c.2780G>T	p.R927L	Missense	LB
**Overall**		8/10	**7/10**	**3/10**												

+, Positive Phenotype; **−**, Negative Phenotype; *NA* Data non-available, *LH* Luteinizing hormone, *FSH* Follicle stimulating hormone, *T* Testosterone, *AMH* anti-Müllerian hormone, *ACTH* Adrenocorticotropic hormone; ^a^ indicating the normal reference range for Inhibin-B, that is 75–350 normal sperm production, 50–80 suspicious spermatogenesis disturbance, <50 spermatogenesis disturbance; ^b^ indicating the parameters were measured at age of 1 years old; ^c^ indicating the parameters were measured at age of 1 year and 10 months; ^d^ indicating the peak value of parameters after gonadotropin hormone releasing hormone (GnRH) stimulated test at age of 2 year and 3 months; ^e^ indicating the peak value of parameters after ACTH stimulated test at age of 2 year and 3 months; ^f^ indicating the parameters were measured at age of 9 months; *ACMG* the American College of Medical Genetics and Genomics, *VUS* Uncertain Significance, *P* Pathogenic, *LB* Likely Benign, *LP* Likely Pathogenic

The clinical features, hormone profiles, and molecular results regarding the 26 previously reported cases are listed in Table 2. Hypospadias was the salient phenotype (22/26), and fourpatients manifested with complete external female genitalia.

**Table 2:** Clinical, biochemical and genetic features of the reported 46, XY DSD patients with MAMLD1 variants

Patient	Visit Age	Phenotype				Serum Hormone		***MAMLD1*** Variants			Functional Studies		Origin
		Hypos-padias	Micro-penis	Crytor-chidism	Urethral Meatus	Gonad Function	Adrenal Function	Nucleotide Change	Amino AcidChange	Variant Type	Transactivat- ion on Hes 3	Protein Expression	
**11**	4 mo	+	**–**	+	Penoscrotal	Normal	NA	c.514G>T	p.E172X	Nonsense	**↓**	**↓**	Japan
**12**	1 mo	**+**	**–**	**–**	Penoscrotal	Normal	NA	c.514G>T	p.E172X	Nonsense	**↓**	**↓**	Japan
**13**	2 yr	**+**	**–**	**–**	Penoscrotal	Normal	NA	c.733C>T	p.Q245X	Nonsense	**↓**	**↓**	Japan
**14**	3 mo	+	**–**	+	Penoscrotal	Normal	NA	c.2176C>T	p.R726X	Nonsense	**↓**	**↓**	Japan
**15**	5.5 yr	**+**	**–**	**–**	Proximal	NA	NA	c.1439 T>C	p.V480A	Missense	NA	NA	USA
**16**^**a**^	2 yr	**+**	**+**	**+**	Penoscrotal	Normal	Normal	c.1439 T>C	p.V480A	Missense	Normal	NA	Spain
**17**	1.2 yr	**+**	**–**	**–**	Penoscrotal	Normal	Normal	c.471delG	p.E157X	Deletion	NA	NA	USA
**18**	1.2 yr	+	**–**	+	Proximal	Normal	Normal	c.471delG	p.E157X	Deletion	NA	NA	USA
**19**	1 mo	**+**	**–**	**–**	Subcoronal	NA	NA	c.1735_1736insCAGCAGCAG	p.579_580insQQQ	Insertion	NA	NA	USA
**20**	NA	+	**–**	**+**	NA	NA	NA	c.1729C>T	p.Q577K	Missense	NA	NA	Sweden
**21**	NA	**+**	**–**	**–**	NA	NA	NA	c.1075C>T	p.P359S	Missense	Normal	NA	Sweden
**22**	NA	**+**	**–**	**–**	NA	NA	NA	c.1842 + 2257A>G		Missense	Normal	NA	Italian
**23**^**a**^	13 yr	Female genitalia			Perineal	–	NA	c.1842 + 2302C>T		Missense	↓	NA	German
**24**	1 mo	**+**	**–**	**–**	Penoscrotal	Normal	Normal	c.1843-6690A>G		splice site	↓	↓	Japanese
**25**	Neonate	**+**	**+**	**–**	Scrotal	Normal	NA	c.428C>A	p.S143X	Nonsense	↓	NA	Caucasian
**26**	3 mo	**+**	**+**	**–**	Perineal	Normal	NA	c.1076C>T	p.P359L	Missense	↓	NA	Caucasian
**27**^**a**^	2 yr	Female genitalia			Perineal	Normal	Normal	c.966C>A	p.H322Q	Missense	Normal	NA	Spain
**28**	2 yr	**+**	**+**	**+**	Penoscrotal	Normal	Normal	c.966C>A	p.H322Q	Missense	Normal	NA	Spain
**29**	NA	**+**	**–**	**–**	Penoscrotal	Normal	NA	c.530C>T	p.T177M	Missense	Normal	NA	Spain
**30**^**a**^	15 d	**+**	**+**	**–**	Penoscrotal	Normal	Normal	c.551delT, c.556G>A	p.L185X, p.D186N	Nonsense	↓	NA	Spain
**31**^b^	70 yr	+	+	**–**	NA	Normal	Normal	c.1430_1431insCAG	p.476_477insQ	Insertion	Normal	NA	Switzerland
**32**	9 mo	**+**	**+**	**–**	Penoscrotal	Normal	Normal	c.1433C>A	p.A478E	Missense	Normal	NA	Spain
**33**	15 mo	**+**	**+**	**–**	Penoscrotal	Normal	Normal	c.2170C>G	p.L724V	Missense	Normal	NA	Spain
**34**^**a**^	12 mo	**+**	**+**	**–**	Penoscrotal	Normal	NA	c.1843-6539G>A		Missense	Normal	NA	Spain
**35**	NA	Female genitalia			Perineal	NA	NA	c.1664A>G	p.Q555R	Missense	Normal	NA	Italian
36	NA	Female genitalia			Perineal	NA	NA	c.1664A>G	p.Q555R	Missense	Normal	NA	Italian

^a^ reared as females; ^b^ Delayed puberty, have fathered a child; NA, Data non-available; +, Positive Phenotype; **−**, Negative Phenotype; ↓, Reduced

### Hormone level measurements

Detailed endocrine data are shown in Table 1. Serum T, LH, and FSH levels were sufficiently high in patients #3 and #6, who were in the mini-puberty period. In addition, a good response of T to hCG stimulation was observed in patients #1–3, #6, and #8. The LH and FSH levels of patient #4 were elevated at 1 year of age but declined with age; at 2 years and 3 months, the basal LH and FSH levels were decreased compared with the normal range, although the peak values after GnRH stimulation were still significantly high, indicating premature reactivation of the hypothalamic GnRH pulse generator/pituitary gonadotropin-gonadal axis. The AMH and INHB levels in patients #1, #3, #6, #8, and #9 were all within the normal range.

Adrenal function was evaluated in patients #1–9. Patients #2, #3, #6, and #8 showed elevated plasma ACTH at the first visit; however, the ACTH levels were detected to be normal without any medication in the follow-up.

As for the 26 previously reported cases, all the measured baseline serum T levels were within the normal range, except in patient #23, who manifested with undetectable T in the serum. Notably, patient #31 had hypospadias, short penis, and delayed puberty, although the basal T level was within the normal range for his age. Because the data for the basal LH and FSH levels were not available, we could not confirm hypogonadism by delayed puberty in case #31. Adrenal function was normal in all the nine cases whose data were available.

### Molecular analysis of the *MAMLD1* gene

In our case series, a total of nine variants were identified, including six missense variants(p.S662R, p.A421P, p.P542S, p.P334S, p.T992I, and p.R927L) and three nonsense variants (p.R356X, p.Q152X, and p.Q124X); p.R356X was identified in two patients. All the variants were inherited from the mothers, and exon 3 was the most commonly affected region (6 out of 9 variations, 67%). According to the ACMG clinical practice guidelines, three variants (p.R356X, p.Q152X, and p.Q124X) in four patients were pathogenic, and variant p.P334S was likely pathogenic, whereas the missense variants p.S662R, p.A421P, and p.T992I are of uncertain significance, and the two missense variants p.P542S and p.R927L are presumably benign.

In the previously reported 26 cases, there were 21*MAMLD1* variants (Table 2). Of these, 15 variants were located on exon 3, and 11 variants were considered pathogenic or likely pathogenic according to the ACMG, whereas the remaining 10 variants were considered as polymorphisms.

### In silico analysis

**Fig. 2:**
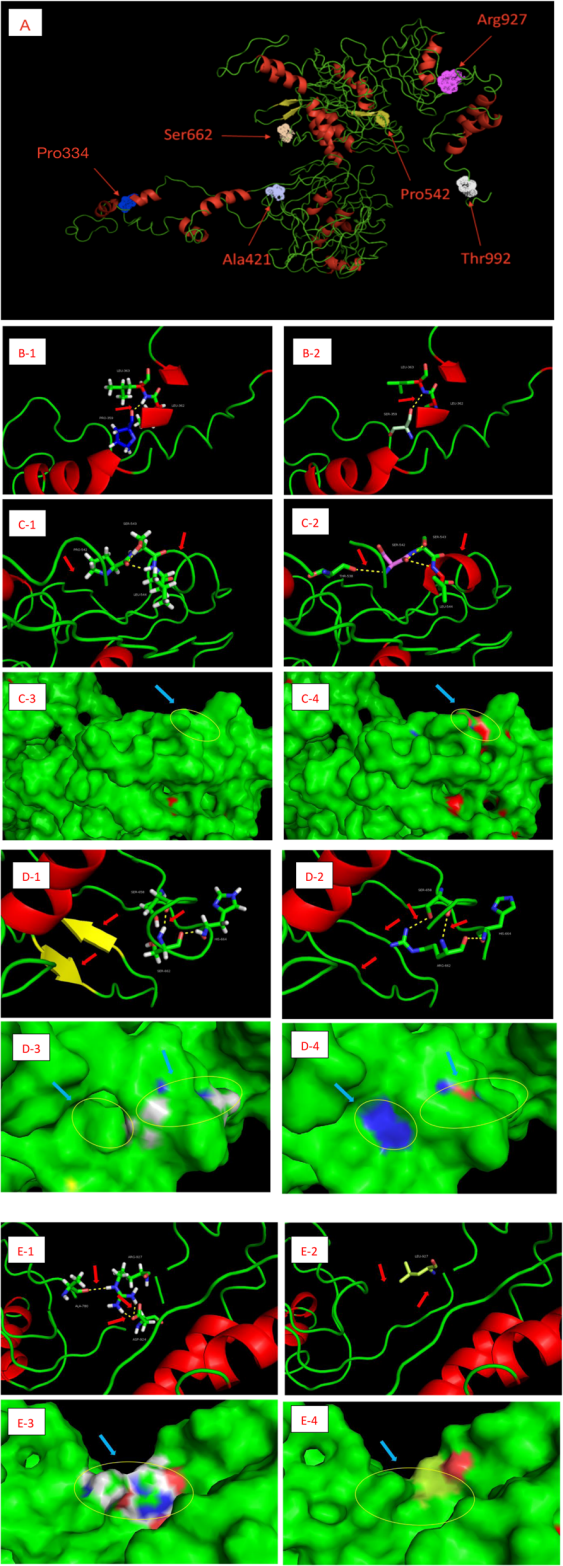
Diagrams of structural models for MAMLD1 (A). Amino-acid residue (dots): p.Pro334Ser is blue,p.Ala421Pro is light gray, p.Pro542Ser is light orange, p.Ser662Arg is light golden, p.Arg927Leu is pink, and p.Thr992Ile is white. Hydrogen bonding by MAMLD1 residues inthe wild type p.Pro359(B-1) and the mutated p.Pro359Ser(B-2) are shown in sticks format with difference signed by red arrows. Hydrogen bonding by MAMLD1 residues inthe wild type p.Pro542(C-1) and the mutated p.Pro542Ser(C-2) are shown in sticks format with difference signed by red arrows. In the visualisation of the predicted molecular surface, detail of the protein surface in the wild type p.Pro542(C-3) and the mutated p.Pro542Ser(C-4) are coloured by CPK notation (red, oxygen; blue, nitrogen; yellow, sulphur; grey, carbon) with distinction signed by light blue arrows and yellow circles. Hydrogen bonding by MAMLD1 residues inthe wild type p.Ser662(D-1) and the mutated p.Ser662Arg(D-2) are shown in sticks format with difference signed by red arrows. Detail of the protein surface in the wild type p.Ser662(D-3) and the mutated p.Ser662Arg(D-4) are coloured by CPK notation with distinction signed by light blue arrows and yellow circles. Hydrogen bonding by MAMLD1 residues inthe wild type p.Arg927(E-1) and the mutated p.Arg927Leu(E-2) are shown in sticks format with difference signed by red arrows. Detail of the protein surface in the wild type p.Arg927(E-3) and the mutated p.Arg927Leu(E-4) are coloured by CPK notation with distinction signed by light blue arrows and yellow circles

The results of the molecular modelling of four selected variants (p.P334S, p.P542S, p.S662R, and p.R927L) are shown in Fig. 2 and are discussed in detail below. Diagrams of structural models for MAMLD1 are shown in Fig. 2A. In the case of both the WT and mutant p.P334S, there is a hydrogen bond between Pro/Ser334 and Leu362 residues in the middle of the two short-chain α-helix regions; however, the polar atoms forming the hydrogen bond are changed, leading to a small perturbance in the structural balance of the protein (Fig. 2B). Compared with the WT, which has two hydrogen bonds formed between Pro542 residue and Ser543 and Leu544 residues, the mutant p.P542S forms a new hydrogen bond between Ser542 and Thr538 residues. Additionally, a part of the nearby coil region is transformed into the short-chain α-helix structure, with the polarity of the protein surface remarkably increased (Fig. 2C). The mutant p.S662R has a hydrogen bond between Arg662 and Ser658 residues in addition to the one between Ser662 and Ser658 residues in the WT. This mutant also has slight changes in the polarity of the protein surface. In addition, the two short-chain β-fold regions in the vicinity are converted into a coil region, with the polarity significantly increased, based on the visualization of the predicted molecular surface (Fig. 2D). In mutant p.R927L, three hydrogen bonds that exist between Arg927 and Ala780 residues, and Arg927 and Asp924 residues in the WT are disrupted. Additionally, the polarity of the protein surface is greatly reduced in this mutant (Fig. 2E). Based on the molecular modelling of the three selected variants (p.P542S, p.S662R, and p.R927L), it is speculated that the mutations lead to a drastic change in the interaction force of the amino acid chain and flexibility of the spatial structure, and such changes may have an effect on the function of the protein.

## Discussion

We have herein described a particular 46,XY DSD case series. All the enrolled patients harboured *MAMLD1* variants, and these patients all shared a broad spectrum of phenotypes. The most prevalent phenotype was hypospadias. The other phenotypes included cryptorchidism, bifid scrotum, and/or micropenis, which is consistent with the phenotypes of previously documented patients with *MAMLD1*-related DSDs (Table 2). Recently, a hemizygous mutation of *MAMLD1*has been reported in a patient with 46,XY complete gonadal dysgenesis, who had complete female external genitalia and primary amenorrhea [17]. In our series, patient#4 showed a progression from precocious to hypergonadotropic hypogonadism, and patient #9 showed isolated micropenis. These phenotypes were reported for the first time in patients with *MAMLD1*-related DSDs, thereby enriching the knowledge of the phenotypes of the disorder.

T levels in the patients were all within the normal range, with mini-puberty being displayed. A good T response after hCG stimulation was observed in all the patients whose data were available. Thus, the present data, in conjunction with the previous findings [10] suggested that *MAMLD1* deficiency caused 46,XY DSD during the foetal life, and it permitted apparently normal T production in infancy until early childhood. However, after observing long-term clinical course in three patients with *MAMLD1* variations, Fukami discovered that long-term *MAMLD1* deficiency might gradually affect the Leydig cell function, resulting in compromised T production [18]. It should be emphasized that case #4 manifested normal male external genitalia at birth but at the age of 1 year, the patient had clinical signs of precocious puberty, such as an increased volume of testes, penis enlargement, as well as the appearance of beard and pubic hair. Hormone investigations revealed elevated LH and FSH levels, indicating a prolonged mini-puberty, which probably results from a mechanism that impairs the prepubertal restraint of gonadotrophin secretion. Two interacting mechanisms have been proposed to explain the physiological restraint of gonadotrophin secretion [19]. One is a gonadal steroid-dependent mechanism, which involves a highly sensitive hypothalamic-pituitary-gonad (HPG) axis negative feedback system. This mechanism has a dominant role in restraining gonadotrophin secretion during the first 3 years of life. The other is a steroid-independent mechanism that involves intrinsic central nervous system inhibition of the hypothalamic GnRH pulse generator. After 3 years of age, the latter plays a critical role. Based on the above theory, a dysfunction may exist in the gonadal steroid negative feedback mechanism in patient#4, indicating primary gonadal dysfunction. This is consistent with the study by Fukami [18], wherein it was proposed that MAMLD1 deficiency may result in primary gonadal dysfunction. The previously reported patient#31 underwent delayed puberty, thereby suggesting partial hypogonadism. The follow-up period of the reported cases, as well as of our cohort, was too short to identify the gonad function in adolescence and adulthood, which will need further longitudinal investigation.

*MAMLD1* had previously been identified as a causative gene for 46,XY DSD. To date, approximately 30 *MAMLD1* sequence variants have been documented in the Human Gene Mutation Database and described in patients who have 46,XY DSD. However, recently, there has been some controversy regarding the causative role of *MAMLD1* gene variations in sex development. In our case series, we analysed the pathogenicity of *MAMLD1* variants in 46,XY DSD patients. A total of nine sequence variants were identified, including six missense and three nonsense variations. All the patients inherited the variants from their mothers, which followed the X-linked recessive inheritance pattern. Three variants (p.R356X, p.T992I and p.P542S) had previously been reported [20], while six variants were reported for the first time (p.Q152X, and p.Q124X, p.P334S, p.S662R, p.A421P, and p.R927L), which contributed to our understanding of this disorder. The difference in variant sites between the reported studies and our cohort may be attributed to ethnic differences.

The three nonsense variants, p.R356X, p.Q152X, and p.Q124X, are all located on exon 3 in line with the findings of the previous studies [8, 10]. Exon 3 encodes the majority (80%) of the functional sequence of the MAMLD1 protein, and skipping this exon would lead to an inactive protein or nonsense-mediated mRNA decay (NMD). The three nonsense variants were predicted to induce an early codon, and thus a short and truncated protein. In addition, they all satisfied the conditions for the occurrence of NMD. Thus, by combining clinical features, inheritance patterns, and biological information, they were considered as pathogenic disease-causing variants.

We identified six missense variants, p.P334S, p.S662R, p.A421P, p.T992I, p.P542S, and p.R927L, in six cases, and no other DSDs or adrenal-related genes were identified in these cases. Based on the in silico analysis results, we speculated that the four variants p.P334S, p.P542S, p.S662R, and p.R927L might lead to a drastic change in the interaction force of the amino acid chain and flexibility of spatial structure, and such changes may have an effect on the function of the protein. Hence, they were considered as pathogenic disease-causing variants.

Molecular modelling of p.A421P and p.T992I suggested that neither of these two variants had an effect on the function of MAMLD1. Clinically, patients with p.A421P manifested with not only precocious puberty, but also hypergonadotropic hypogonadism, and patients with p.T992I presented isolated micropenis. None of these patients manifested hypospadias; hence, these variants might be considered as polymorphisms. The polymorphisms of the genes implicated in penile development, testis differentiation, and/or hormone actions have been discussed as susceptibility factors for hypospadias [21–23]. Thus, the implication of *MAMLD1* in the occurrence of 46,XY DSD may also involve gene polymorphisms. Several studies have reported the involvement of *MAMLD1* polymorphisms in hypospadias patients [21–24]. The mechanism by which *MAMLD1* polymorphisms would modulate the risk of hypospadias may include compromised T production during the critical period of sex differentiation, as well as damage caused by the structural instability of the resultant protein, and altered surface accessibility of the protein compared with its WT counterpart. Moreover, the T production during the critical period may be worsened by environmental disruptors, suggesting that *MAMLD1* polymorphisms may modulate an individual’s susceptibility to environmental factors.

Most of the previously reported cases manifested features similar to those of our case series, and exon 3 was also most commonly affected in this case-cohort. Functional studies have shown that some *MAMLD1*variantshave normal *Hes3* transactivation function, and some are also presented in the normal population. Animal experiments have shown that mice with *MAMLD1* deficiency present with normal genital and reproductive development, and this observation is different from that observed in patients with *MAMLD1* variants, suggesting that the role of *MAMLD1* may differ between mice and humans. Accordingly, investigation of more cases is essential for a better understanding of the pathological mechanisms of the variants of this gene.

There are several limitations to this study. First, the sample size was too small to establish a relationship between genotypes and phenotypes, thus diminishing the significance of the observations. Second, the follow-up period was too short to monitor the testis function through time, especially in late childhood. Third, apart from the previously reported *MAMLD1* variants, the effect of the novel *MAMLD1* variants in our cohort on the development of hypospadias needs to be confirmed by further functional genetic studies. Therefore, our future work will involve an increased sample size and a more extended follow-up period. In addition, functional studies will be performed to further clarify the exact mechanism in 46,XY DSD patients harbouring*MAMLD1* variations.

## Conclusion

Our case series indicates that 46,XY DSD patients harbouring*MAMLD1*variants manifest a broad spectrum of phenotypes, with the majority showing hypospadias; other phenotypes included cryptorchidism, bifid scrotum, and/or micropenis. Central precocious puberty may also be manifested and should not be ignored. The testicular function may be gradually impaired with age. Thus, close monitoring of LH, FSH, and T levels is of great importance. Variants often occurred in exon 3. The nine novel reported variants enriched the mutation database and contributed to our understanding of the pathophysiological process of 46,XY DSDs.

## Data Availability

Data are available in a public, open access repository. All the data relevant to the study are included in the article or uploaded as supplementary information.

## Abbreviations

DSD: Disorders of sex development
MAMLD1: Mastermind-like domain-containing 1
CXorf6: Chromosome X open reading frame 6
SF-1: Steroidogenic factor-1
WT: Wild-type
NGS: Next-generation sequencing
WES: Whole-exome sequencing
T: Testosterone
LH: Luteinizing hormone
FSH: Follicle-stimulating hormone
GnRH: Gonadotropin-releasing hormone
hCG: Human chorionic gonadotropin
AMH: Anti-Müllerian hormone
INHB: Inhibin-B
ACTH: Adrenocorticotropic hormone
ACMG: American College of Medical Genetics and Genomics
NMD: Nonsense-mediated mRNA decay
